# 3,3′-(Ethane-1,2-diyl)bis(6-meth­oxy-3,4-dihydro-2*H*-1,3-benzoxazine) mono­hydrate

**DOI:** 10.1107/S1600536812035519

**Published:** 2012-08-23

**Authors:** Augusto Rivera, Jairo Camacho, Jaime Ríos-Motta, Monika Kučeraková, Michal Dušek

**Affiliations:** a Universidad Nacional de Colombia, Sede Bogotá, Facultad de Ciencias, Departamento de Química, Cra 30 No. 45-03, Bogotá, Código Postal 111321, Colombia; b Institute of Physics ASCR, v.v.i., Na Slovance 2, 182 21 Praha 8, Czech Republic

## Abstract

The asymmetric unit of the title compound, C_20_H_24_N_2_O_4_·H_2_O, contains one half-organic mol­ecule (an inversion centre generates the other half of the mol­ecule) and a half-mol­ecule of water (the O atom has site symmetry 2). The near planarity of the fused-benzene ring is illustrated by the very small deviations of all the atoms from the plane [largest deviation = 0.0092 (11) Å. The six-membered N,O-containing ring adopts a half-chair conformation. The observed N—CH_2_ and CH_2_—O bond lengths can be correlated to the manifestation of an anomeric effect in the N—CH_2_—O unit. In the crystal, the mol­ecules are connected into zigzag chains parallel to [001] through O—H⋯N hydrogen bonds formed between the oxazinic N atom and the solvent water mol­ecule. The chains are consolidated by C—H⋯O inter­actions.

## Related literature

For related structures, see: Rivera *et al.* (2012 ▶, 2011 ▶, 2010 ▶). For the preparation of the title compound, see: Rivera *et al.* (1989 ▶). For ring conformations, see: Cremer & Pople (1975 ▶). For bond-length data, see: Allen *et al.* (1987 ▶).
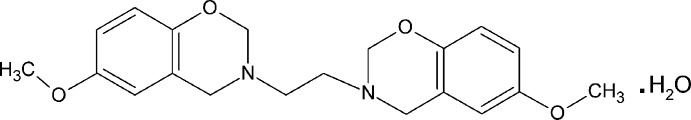


## Experimental

### 

#### Crystal data

C_20_H_24_N_2_O_4_·H_2_O
*M* *_r_* = 374.4
Monoclinic, 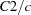

*a* = 30.2999 (9) Å
*b* = 5.2132 (2) Å
*c* = 11.6058 (4) Å
β = 91.153 (2)°
*V* = 1832.87 (11) Å^3^
*Z* = 4
Cu *K*α radiation
μ = 0.80 mm^−1^
*T* = 120 K
0.17 × 0.13 × 0.13 mm

#### Data collection

Oxford Diffraction Xcalibur Atlas Gemini ultra diffractometer
Absorption correction: multi-scan (*CrysAlis PRO*; Agilent, 2012 ▶) *T*_min_ = 0.104, *T*_max_ = 1
18257 measured reflections
1639 independent reflections
1440 reflections with *I* > 3σ(*I*)
*R*_int_ = 0.033

#### Refinement

*R*[*F* ^2^ > 2σ(*F* ^2^)] = 0.030
*wR*(*F* ^2^) = 0.094
*S* = 1.75
1639 reflections
127 parameters
H atoms treated by a mixture of independent and constrained refinement
Δρ_max_ = 0.17 e Å^−3^
Δρ_min_ = −0.14 e Å^−3^

### 

Data collection: *CrysAlis PRO* (Agilent, 2012 ▶); cell refinement: *CrysAlis PRO*; data reduction: *CrysAlis PRO*; program(s) used to solve structure: *Superflip* (Palatinus & Chapuis, 2007 ▶); program(s) used to refine structure: *JANA2006* (Petříček *et al.*, 2006 ▶); molecular graphics: *DIAMOND* (Brandenburg & Putz, 2005 ▶); software used to prepare material for publication: *JANA2006*.

## Supplementary Material

Crystal structure: contains datablock(s) global, I. DOI: 10.1107/S1600536812035519/bx2424sup1.cif

Structure factors: contains datablock(s) I. DOI: 10.1107/S1600536812035519/bx2424Isup2.hkl

Supplementary material file. DOI: 10.1107/S1600536812035519/bx2424Isup3.cml

Additional supplementary materials:  crystallographic information; 3D view; checkCIF report

## Figures and Tables

**Table 1:** Hydrogen-bond geometry (Å, °)

*D*—H⋯*A*	*D*—H	H⋯*A*	*D*⋯*A*	*D*—H⋯*A*
C4—H1*c*4⋯O1^i^	0.96	2.46	3.2982 (13)	145.91
O3—H1*o*3⋯N1^ii^	0.914 (15)	2.076 (15)	2.9870 (12)	174.8 (13)

Symmetry codes: (i) 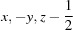; (ii) 
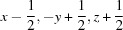
.

**Fig. 1.**
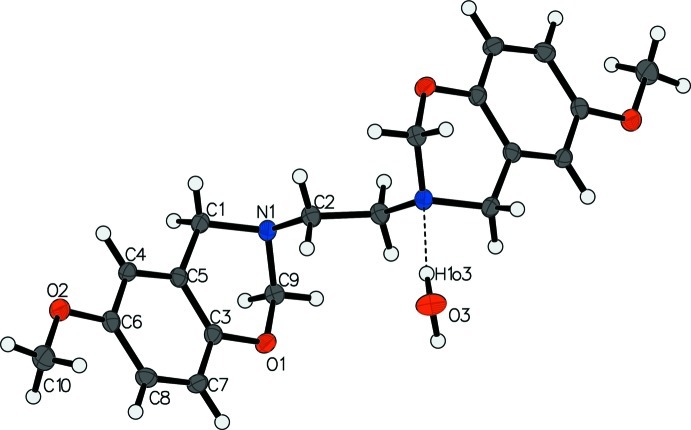
A view of (I) with the numbering scheme. Displacement ellipsoids are drawn at the 50% probability level and H atoms are shown as small spheres of arbitrary radii. In the organic molecule, the labelled atoms are related with unlabelled atoms by symmetry code [1-x, 1-y, 1-z]. In the water molecule the labelled atom is related with unlabelled atom by symmetry code [1-x,y,3/2-z]. The hydrogen bond interaction is shown as dashed lines.

**Fig. 2.**
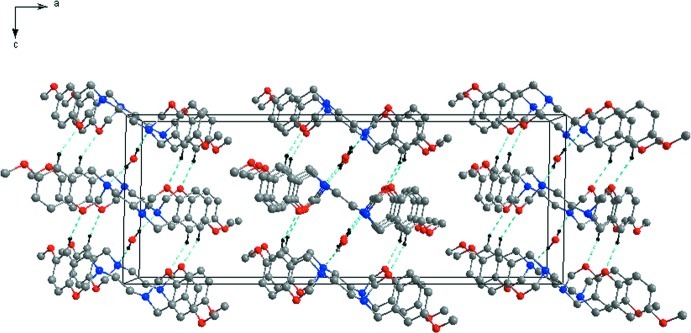
Packing of the molecules of the title compound view along the b axis. Intermolecular hydrogen bonds are shown as dashed lines. For clarity only shows the H atoms involved in hydrogen bonds.

## supplementary crystallographic information

### Comment

As models to examine the anomeric effect we have recently studied the influence of substituent such as chlorine and methyl on 3,3'-ethane-1,2-diylbis(3,4-dihydro-2*H*-1,3-benzoxazine) (Rivera, *et al.*, 2010; 2011; 2012). In order to continue this research, we have synthesized the title compound and obtained suitable crystals for single-crystal X-ray diffraction analysis.

The asymmetric unit of the title compound (Fig. 1), C_20_H_24_N_2_O_4_.H_2_O, contains one half-organic molecule (an inversion centre generates the other half of the molecule) and one half water molecule The planarity of the fused-benzene ring is illustrated by very small deviation of all the atoms from these planes [largest deviations = 0.0092 (11) Å for C-3]. The half-chair conformation, with puckering parameters Q = 0.512 (2) Å, θ =129.6 (2)°, φ =283.6 (3)° (Cremer & Pople, 1975), of the six-membered N,*O*-containing ring is analyzed with respect to the plane formed by O1/C3/C5/C1 and the corresponding deviations of the atoms C9 and N1 are 0.3002 (11) and 0.3350 (10) Å, respectively. The methoxy substituent at the C6 atom forms the torsion angle of 2.63 (14) ° [synperiplanar conformation] with the atom set O2/C6/C8/C7. The bond lengths and angles are within normal ranges, whereas the C9—O1 bond length [1.4463 (13) Å] is shorter than the corresponding C—O bonds found in the chlorine related structure [1.529 (2) Å] (Rivera *et al.*, 2010), whereas the N1—C9 bond length of [1.4445 (14) Å] is longer than the corresponding N—C bonds found in the related structure [1.3690 (16) Å] (Rivera *et al.*, 2010). In contrast to the chloro analog, the title compound was found to be more agreement with other related structures (Rivera *et al.*, 2012, 2011), and with the normal values for O—CH_2_ (1.470) and CH_2_—N (1.469) (Allen *et al.*, 1987), indicating that the methoxy substituent decrease the influence of stereoelectronic effects in the N—CH_2_—O moiety.

In the crystal structure, the molecules of the title compound are conected into *zigzag* chains parallel to [001] through O—H···N hydrogen bonds formed between its oxazinic N atom and the solvent water molecule. This chain is further stabilized by weak C—H···O hydrogen bonding interactions between H4 and O1. (Figure 2)

### Experimental

The title compound was synthesized according to the literature procedure (Rivera *et al.*,1989), and the single crystals were obtained by slow evaporation from a ethanol/water solvent mixture at room temperature.

### Refinement

All hydrogen atoms were discernible in difference Fourier maps and could be refined to reasonable geometry. According to common practice the hydrogen atoms attached to carbons were kept in ideal positions with C–H distance 0.96%A during the refinement. The methyl H atoms were allowed to rotate freely about the adjacent C—C bonds. The coordinates of the hydrogen atom bonded to oxygen were refined freely. All H atoms were refined with displacement coefficients *U*_iso_(H) set to 1.5*U*_eq_(C, O) for the methyl- and hydroxyl groups and to 1.2Ueq(C) for the CH–, and CH_2_– groups.

### Crystal data

**Table:**

C_20_H_24_N_2_O_4_·H_2_O	*F*(000) = 800
*M**_r_* = 374.4	*D*_x_ = 1.357 Mg m^−^^3^
Monoclinic, *C*2/*c*	Cu *K*α radiation, λ = 1.5418 Å
Hall symbol: -C 2yc	Cell parameters from 10577 reflections
*a* = 30.2999 (9) Å	θ = 2.9–67.0°
*b* = 5.2132 (2) Å	µ = 0.80 mm^−^^1^
*c* = 11.6058 (4) Å	*T* = 120 K
β = 91.153 (2)°	Prism, white
*V* = 1832.87 (11) Å^3^	0.17 × 0.13 × 0.13 mm
*Z* = 4	

### Data collection

**Table:**

Oxford Diffraction Xcalibur Atlas Gemini ultra diffractometer	1639 independent reflections
Radiation source: Enhance Ultra (Cu) X-ray Source	1440 reflections with *I* > 3σ(*I*)
Mirror monochromator	*R*_int_ = 0.033
Detector resolution: 10.3784 pixels mm^-1^	θ_max_ = 67.2°, θ_min_ = 2.9°
ω scans	*h* = −36→36
Absorption correction: multi-scan (*CrysAlis PRO*; Agilent, 2012)	*k* = −6→6
*T*_min_ = 0.104, *T*_max_ = 1	*l* = −13→13
18257 measured reflections	

### Refinement

**Table:**

Refinement on *F*^2^	H atoms treated by a mixture of independent and constrained refinement
*R*[*F*^2^ > 2σ(*F*^2^)] = 0.030	Weighting scheme based on measured s.u.'s *w* = 1/(σ^2^(*I*) + 0.0016*I*^2^)
*wR*(*F*^2^) = 0.094	(Δ/σ)_max_ = 0.015
*S* = 1.75	Δρ_max_ = 0.17 e Å^−^^3^
1639 reflections	Δρ_min_ = −0.14 e Å^−^^3^
127 parameters	Extinction correction: B-C type 1 Gaussian isotropic (Becker & Coppens, 1974)
0 restraints	Extinction coefficient: 1300 (300)
49 constraints	

### Special details

**Table:**

Refinement. The refinement was carried out against all reflections. The conventional *R*-factor is always based on *F*. The goodness of fit as well as the weighted *R*-factor are based on *F* and *F*^2^ for refinement carried out on *F* and *F*^2^, respectively. The threshold expression is used only for calculating *R*-factors *etc*. and it is not relevant to the choice of reflections for refinement.The program used for refinement, Jana2006, uses the weighting scheme based on the experimental expectations, see _refine_ls_weighting_details, that does not force *S* to be one. Therefore the values of *S* are usually larger than the ones from the *SHELX* program.

### Fractional atomic coordinates and isotropic or equivalent isotropic displacement parameters (Å^2^)

**Table:**

	*x*	*y*	*z*	*U*_iso_*/*U*_eq_	
O1	0.90016 (2)	0.11497 (15)	0.06702 (6)	0.0199 (2)	
O2	0.80451 (3)	−0.57728 (15)	−0.19811 (7)	0.0218 (3)	
O3	0.5	0.0044 (2)	0.25	0.0278 (4)	
N1	0.95328 (3)	0.15870 (18)	−0.08373 (7)	0.0175 (3)	
C1	0.92150 (3)	0.0638 (2)	−0.17210 (9)	0.0187 (3)	
C2	0.98132 (3)	−0.0506 (2)	−0.03816 (9)	0.0185 (3)	
C3	0.87593 (3)	−0.0523 (2)	−0.00091 (9)	0.0170 (3)	
C4	0.85915 (3)	−0.2627 (2)	−0.18039 (9)	0.0170 (3)	
C5	0.88428 (3)	−0.0852 (2)	−0.11834 (9)	0.0164 (3)	
C6	0.82677 (3)	−0.4068 (2)	−0.12743 (9)	0.0177 (3)	
C7	0.84416 (4)	−0.1983 (2)	0.05238 (9)	0.0198 (3)	
C8	0.81931 (4)	−0.3764 (2)	−0.01008 (10)	0.0200 (3)	
C9	0.92843 (3)	0.2868 (2)	0.00424 (9)	0.0179 (3)	
C10	0.77298 (4)	−0.7400 (2)	−0.14564 (10)	0.0260 (4)	
H1c1	0.936552	−0.044083	−0.225599	0.0225*	
H2c1	0.909537	0.206326	−0.214813	0.0225*	
H1c2	0.993326	−0.145584	−0.101079	0.0222*	
H2c2	0.963614	−0.167436	0.005101	0.0222*	
H1c4	0.864263	−0.286099	−0.261076	0.0205*	
H1c7	0.839223	−0.176396	0.13323	0.0237*	
H1c8	0.79727	−0.477366	0.027191	0.024*	
H1c9	0.948384	0.371178	0.05716	0.0215*	
H2c9	0.910924	0.420868	−0.030257	0.0215*	
H1c10	0.76001	−0.851305	−0.20284	0.0312*	
H2c10	0.787328	−0.841082	−0.086776	0.0312*	
H3c10	0.750337	−0.637226	−0.111909	0.0312*	
H1o3	0.4874 (5)	0.112 (3)	0.3020 (13)	0.0334*	

### Atomic displacement parameters (Å^2^)

**Table:**

	*U*^11^	*U*^22^	*U*^33^	*U*^12^	*U*^13^	*U*^23^
O1	0.0221 (4)	0.0216 (5)	0.0160 (4)	−0.0038 (3)	0.0011 (3)	−0.0019 (3)
O2	0.0213 (4)	0.0237 (5)	0.0205 (4)	−0.0064 (3)	−0.0002 (3)	−0.0021 (3)
O3	0.0369 (7)	0.0197 (7)	0.0274 (6)	0	0.0112 (5)	0
N1	0.0171 (4)	0.0186 (5)	0.0167 (4)	−0.0008 (4)	−0.0019 (3)	−0.0001 (4)
C1	0.0183 (5)	0.0233 (6)	0.0146 (5)	−0.0022 (5)	−0.0014 (4)	0.0015 (4)
C2	0.0173 (5)	0.0175 (6)	0.0208 (6)	0.0007 (4)	−0.0013 (4)	−0.0009 (4)
C3	0.0168 (5)	0.0171 (6)	0.0171 (6)	0.0027 (4)	−0.0018 (4)	−0.0008 (4)
C4	0.0174 (5)	0.0197 (6)	0.0140 (5)	0.0027 (4)	−0.0007 (4)	0.0007 (4)
C5	0.0151 (5)	0.0175 (6)	0.0166 (5)	0.0025 (4)	−0.0010 (4)	0.0034 (4)
C6	0.0161 (5)	0.0173 (6)	0.0196 (6)	0.0017 (4)	−0.0026 (4)	0.0001 (4)
C7	0.0209 (5)	0.0235 (6)	0.0150 (5)	0.0018 (5)	0.0022 (4)	0.0008 (4)
C8	0.0182 (5)	0.0212 (6)	0.0208 (6)	−0.0011 (4)	0.0031 (4)	0.0033 (4)
C9	0.0179 (5)	0.0162 (6)	0.0195 (5)	−0.0004 (4)	−0.0004 (4)	0.0000 (4)
C10	0.0249 (6)	0.0251 (7)	0.0279 (6)	−0.0096 (5)	−0.0007 (5)	0.0007 (5)

### Geometric parameters (Å, º)

**Table:**

O1—C3	1.3776 (13)	C3—C5	1.4016 (15)
O1—C9	1.4463 (13)	C3—C7	1.3827 (15)
O2—C6	1.3765 (13)	C4—C5	1.3902 (15)
O2—C10	1.4234 (15)	C4—C6	1.3890 (15)
O3—H1o3	0.914 (15)	C4—H1c4	0.96
O3—H1o3^i^	0.914 (15)	C6—C8	1.3941 (15)
N1—C1	1.4780 (13)	C7—C8	1.3903 (16)
N1—C2	1.4743 (14)	C7—H1c7	0.96
N1—C9	1.4445 (14)	C8—H1c8	0.96
C1—C5	1.5143 (15)	C9—H1c9	0.96
C1—H1c1	0.96	C9—H2c9	0.96
C1—H2c1	0.96	C10—H1c10	0.96
C2—C2^ii^	1.5181 (14)	C10—H2c10	0.96
C2—H1c2	0.96	C10—H3c10	0.96
C2—H2c2	0.96		
			
C3—O1—C9	114.70 (8)	C1—C5—C3	119.23 (9)
C6—O2—C10	117.04 (8)	C1—C5—C4	122.19 (9)
H1o3—O3—H1o3^i^	104.2 (14)	C3—C5—C4	118.52 (10)
C1—N1—C2	111.35 (9)	O2—C6—C4	115.30 (9)
C1—N1—C9	107.66 (8)	O2—C6—C8	124.65 (10)
C2—N1—C9	113.16 (8)	C4—C6—C8	120.04 (10)
N1—C1—C5	111.48 (8)	C3—C7—C8	120.60 (10)
N1—C1—H1c1	109.47	C3—C7—H1c7	119.7
N1—C1—H2c1	109.47	C8—C7—H1c7	119.7
C5—C1—H1c1	109.47	C6—C8—C7	119.25 (10)
C5—C1—H2c1	109.47	C6—C8—H1c8	120.38
H1c1—C1—H2c1	107.39	C7—C8—H1c8	120.38
N1—C2—C2^ii^	111.70 (9)	O1—C9—N1	113.10 (9)
N1—C2—H1c2	109.47	O1—C9—H1c9	109.47
N1—C2—H2c2	109.47	O1—C9—H2c9	109.47
C2^ii^—C2—H1c2	109.47	N1—C9—H1c9	109.47
C2^ii^—C2—H2c2	109.47	N1—C9—H2c9	109.47
H1c2—C2—H2c2	107.15	H1c9—C9—H2c9	105.58
O1—C3—C5	121.97 (9)	O2—C10—H1c10	109.47
O1—C3—C7	117.40 (9)	O2—C10—H2c10	109.47
C5—C3—C7	120.56 (10)	O2—C10—H3c10	109.47
C5—C4—C6	121.01 (10)	H1c10—C10—H2c10	109.47
C5—C4—H1c4	119.49	H1c10—C10—H3c10	109.47
C6—C4—H1c4	119.5	H2c10—C10—H3c10	109.47

Symmetry codes: (i) −*x*+1, *y*, −*z*+1/2; (ii) −*x*+2, −*y*, −*z*.

### Hydrogen-bond geometry (Å, º)

**Table:**

*D*—H···*A*	*D*—H	H···*A*	*D*···*A*	*D*—H···*A*
C4—H1*c*4···O1^iii^	0.96	2.46	3.2982 (13)	145.91
O3—H1*o*3···N1^iv^	0.914 (15)	2.076 (15)	2.9870 (12)	174.8 (13)

Symmetry codes: (iii) *x*, −*y*, *z*−1/2; (iv) *x*−1/2, −*y*+1/2, *z*+1/2.
